# Susceptibility of *ApoB* and *PCSK9* Genetic Polymorphisms to Diabetic Kidney Disease Among Chinese Diabetic Patients

**DOI:** 10.3389/fmed.2021.659188

**Published:** 2021-04-06

**Authors:** Liang Ma, Shaoting Wang, Hailing Zhao, Meijie Yu, Xiangling Deng, Yongwei Jiang, Yongtong Cao, Ping Li, Wenquan Niu

**Affiliations:** ^1^Clinical Laboratory, China-Japan Friendship Hospital, Beijing, China; ^2^Beijing Key Laboratory of Immune-Mediated Inflammatory Diseases, Institute of Clinical Medical Science, China-Japan Friendship Hospital, Beijing, China; ^3^Department of Nephrology, China-Japan Friendship Hospital, Beijing, China; ^4^Institute of Clinical Medical Science, China-Japan Friendship Hospital, Beijing, China

**Keywords:** diabetic kidney disease, single nucleotide polymorphism, association, *ApoB*/*PCSK9* genes, risk

## Abstract

This study aimed to investigate the susceptibility of 8 polymorphisms in *ApoB* and *PCSK9* genes to diabetic kidney disease (DKD) in Chinese patients with type 2 diabetes mellitus. This is a case-control association study, including 575 DKD cases and 653 controls. Genotypes were determined using ligase detection reaction method, and data are analyzed using STATA software. The genotype distributions of rs1042034 and rs12720838 differed significantly between the two groups (*P* < 0.001 and *P* = 0.008, respectively). After adjusting for confounding factors, the mutations of rs1042034 and rs12720838 were associated with the significantly increased risk of DKD. For instance, carriers of rs1042034 T allele (CT and TT genotypes) were 1.07 times more likely to have DKD than carriers of rs1042034 CC genotype [odds ratio (OR) = 1.07, 95% confidence interval (CI): 1.03–1.10, *P* < 0.001]. Further, haplotype T-A-G-T in *ApoB* gene was overrepresented in cases (18.10%) compared with controls (12.76%) (P_Simulated_ = 0.045), and haplotype T-A-G-T was associated with a 33% increased risk of DKD (OR = 1.33, 95% CI: 1.04, 1.70). In further haplotype-phenotype analysis, significant association was only noted for hypertension and omnibus haplotypes in *ApoB* gene (P_Simulated_ = 0.001). Our findings indicate that *ApoB* gene is a candidate gene for DKD in Chinese patients with type 2 diabetes mellitus.

## Introduction

As a major microvascular complication of diabetes mellitus, diabetic kidney disease (DKD) has skyrocketed to epidemic proportions. Latest statistics indicates that the age-standardized prevalence of DKD worldwide was 15.48/1,000 and 16.50/1,000 in men and women, respectively (1). Although global DKD prevalence has remained stabilized during the last three decades, the mortality rate of DKD is growing in comparison to that of other types of chronic kidney disease (2). Effective strategies should be developed to curb this global burden (3). A practical strategy is the identification of possible risk factors to potentially predict subjects who are more likely to pre-dispose to DKD, thereby helping doctors and healthcare professionals to make immediate prevention and control measures against this disease.

It is widely accepted that DKD is a complex multifactorial disease, partly under genetic control (4). Previous evidence revealed that DKD occurs in familial clusters, indicating a strong heritable component in the pathogenesis (5, 6). The results of recently completed genome-wide association studies have advanced knowledge on the genetic underpinnings of DKD (7–10). Despite much endeavors, the causal genetic determinants of DKD are not yet fully understood. As such, candidate gene approach represents an alternative strategy by focusing on the genes with strong biological or clinical implications (11). The genes encoding apolipoprotein B (ApoB) and proprotein convertase subtilisin/kexin type 9 (PCSK9) are such candidate genes for DKD. Clinical studies have shown that circulating ApoB served as an independent predictor of renal replacement therapy in DKD patients (12, 13), and PCSK9 can promote hypercholesterolemia, and PCSK9 concentrations were associated with chronic kidney disease stages (14) and lipid-lowering regimens (15). However, in the medical literature, the genetic pre-disposition of *ApoB* and *PCSK9* genes to DKD is rarely reported.

To fill this gap in knowledge and yield more information for future research, we genotyped 5 single nucleotide polymorphisms (SNPs) in *ApoB* gene and 3 SNPs in *PCSK9* gene in 575 DKD patients and 653 controls, and investigated their susceptibility to DKD risk, both individually and jointly.

## Methods

### Study Design and Ethical Approval

This is a case-control association study officially approved by the institutional review boards of the China-Japan Friendship Hospital. All study subjects were enrolled from this hospital during the period between August 2016 and December 2018, and they gave written informed consent prior to drawing blood samples for genetic analysis and filling out questionnaires.

### Study Subjects

A total of 1,228 patients, who were diagnosed with type 2 diabetes mellitus, were enrolled in this study. Of these patients, 575 had newly-diagnosed and histologically-confirmed DKD, as the case group. The remaining 653 patients who had experienced type 2 diabetes mellitus for 7 or more years, were clinically confirmed to be free of DKD, and had no history of severe kidney diseases formed the control group.

### Eligibility Criteria

DKD was diagnosed according to the National Kidney Foundation Kidney Disease Outcomes Quality Initiative (NKF-K/DOQI) guidelines (16).

In detail, subjects in the case group were included if they had a clinical diagnosis of type 2 diabetes mellitus and 24h urinary albumin >500 mg/24 h or an albumin creatinine ratio (ACR) >30 mg/g, and subjects were excluded if they had no previous history of kidney diseases, or if they had primary or secondary kidney diseases that caused proteinuria, such as IgA nephropathy, membranous nephropathy, lupus nephritis, obstructive renal disease, and acute urinary tract infection.

Subjects in the control group were included if they had a clinical diagnosis of type 2 diabetes mellitus and ACR < 30 mg/g. Exclusion criteria were identical as the case group.

### Demographic Data Collection

A structured questionnaire was designed to collect information on age, gender, body weight/height, cigarette smoking, hypertension status, and duration of type 2 diabetes mellitus. Body mass index (BMI) was calculated as body weight (in kilometers) divided by body height (in meters) squared.

### Laboratory Biomarker Measurement

Laboratory markers included 24 h urinary albumin excretion, ACR, high density lipoprotein cholesterol (HDLC), low density lipoprotein cholesterol (LDLC), total cholesterol (TC), triglyceride (TG), hemoglobin A1c (HbA1c), and homocysteine (HCY). Serum concentrations of fasting TG, TC, HDLC, LDLC, and HCY were measured using an automated biochemical analyzer (AU5800 Clinical Chemistry System, Beckman Coulter, Brea, CA, USA). HBA1c was measured using the D-10 Hemoglobin Testing System (Bio-Rad, Hercules, CA, USA).

### Genomic DNA Extraction and Genotyping

Genomic DNA was extracted from whole blood cells according to the manufacturer instructions, and quantified using the NanoDrop 1000 spectrophotometer (ThermoScientific). DNA samples were frozen at −20°C until mass genotyping.

Five SNPs in *ApoB* gene, including rs1042034, rs679899, rs676210, rs1367117, rs12720838, and three SNPs in *PCSK9* gene, including rs662145, rs45448095, rs11583680, were genotyped by use of the ligase detection reaction (LDR) method.

In detail, 50 ng DNA was amplified in 15 μl reaction mixture containing 7.5 μL of Premix Ex Taq, and 10 pmol of each primer for the amplification of genomic sequences. Pre-heating of the mixture at 94°C for 3 min followed by 35 cycles of denaturation at 94°C for 15 s, annealing at 55°C for 30 s, and elongation at 75°C for 90 s. LDR ligation reaction was in 10 μl reaction mixture containing 3 μl of polymerase chain reaction (PCR) product, 1 μl of 10^*^ Taq DNA ligase buffer, 0.125 μl of Taq DNA ligase, and 0.02 μl of mix probes.

The primer sequences and probe sequences of each SNP are summarized in Supplementary Tables 1, 2, respectively.

### Statistical Analysis

Continuous data are expressed as median (interquartile range), and categorical data as percentage. Two-group comparisons were completed using the Wilcoxon rank-sum test or χ^2^-test when appropriate. The genotypes and alleles of each SNP under study, as well as the tests for Hardy-Weinberg equilibrium, were compared using χ^2^-test between controls and cases. Risk prediction of each SNP for DKD, summarized as odds ratio (OR) and 95% confidence interval (CI), was calculated separately under additive and dominant models before and after adjusting for confounding factors. Linkage patterns of SNPs under study in each gene were examined using the HaploView software (version 4.2, Cambridge, MA, USA).

The frequencies of derived haplotypes were estimated using the Haplo.Stats program in the R software (version 3.6.1). Comparison of derived haplotypes between the two groups, prediction estimates of each haplotype for DKD risk, and association of omnibus haplotypes with baseline characteristics were completed using the Haplo.Stats program as well.

Unless otherwise stated, statistical analyses were performed using the STATA software (version 14.1, Stata Corp., College Station, TX). The power to detect statistical significance was derived using the PS Power and Sample Size Calculations (version 3.0) (17).

## Results

### Baseline Characteristics

Table 1 shows the baseline characteristics of study subjects. Cases were significantly older than controls (median age, 62 vs. 60 years, *P* = 0.001). There was a slightly high proportion of males in cases relative to controls (66.4% vs. 60.8, *P* = 0.041), as well as a high proportion of smokers (*P* = 0.041) and hypertension (*P* < 0.001).

**Table 1:** The baseline characteristics of the study subjects.

**Characteristics**	**Controls (*n* = 653)**	**Cases (*n* = 575)**	***P***
Age, years	60 (53, 67)	62 (54, 70)	0.001
Males, %	60.8	66.4	0.041
Smokers, %	32.0	37.6	0.041
Hypertension, %	51.6	78.1	<0.001
BMI, kg/m^2^	25.31 (23.40, 27.70)	25.78 (24.00, 28.39)	0.003
Duration of diabetes, years	14 (10, 18)	15 (9, 21)	0.208
TC, mmol/L	4.15 (3.50, 4.88)	4.19 (3.43, 5.03)	0.715
HbA1C, mmol/L	7.85 (6.80, 9.20)	7.80 (6.70, 9.30)	0.984
HCY, μmol/L	11.10 (9.41, 13.26)	13.37 (10.74, 16.81)	<0.001
TG, mmol/L	1.45 (1.03, 2.18)	1.65 (1.13, 2.44)	<0.001
HDLC, mmol/L	0.99 (0.83, 1.20)	0.97 (0.79, 1.19)	1.578
LDLC, mmol/L	2.44 (1.93, 3.06)	2.39 (1.83, 3.08)	1.768

*BMI, body mass index; SBP, systolic blood pressure; DBP, diastolic blood pressure; TC, total cholesterol; HbA1C, hemoglobin A1c; HCY, homocysteine; TG, triglyceride; HDLC, high-density lipoprotein cholesterol; LDLC, low-density lipoprotein cholesterol. Data are expressed as median (interquartile range) or percentage, when appropriate. P was calculated using the Wilcoxon rank-sum test for continuous data and the χ^2^-test for categorical data*.

### Linkage Disequilibrium

In *ApoB* gene, two SNPs, rs1042034 and rs676210, were in complete linkage, and in *PCSK9* gene, rs45448095 and rs11583680 were in complete linkage. As such, rs676210 and rs11583680 were removed from the following analyses.

### Genotype and Allele Distributions

The genotype and allele distributions of the remaining 6 SNPs between controls and cases are presented in Table 2. The genotype distributions of rs1042034 and rs12720838 differed significantly between the two groups (*P* < 0.001 and *P* = 0.008, respectively). No significance was noted for the comparisons of the other SNPs. The genotype distributions of all study SNPs satisfied the Hardy-Weinberg equilibrium at a significance level of 10%.

**Table 2:** The genotype and allele distributions of studied polymorphisms between controls and cases.

**SNPs**	**Genotype/allele**	**Controls**	**Cases**	**χ^**2**^**	P^*^
***ApoB*** **gene**
rs1042034	CC	373 (57.12%)	319 (55.48%)	18.11	<0.001
	CT	270 (41.35%)	221 (38.43%)		
	TT	10 (1.53%)	35 (6.09%)		
	T	290 (22.21%)	291 (25.30%)	3.25	0.071
rs679899	AA	453 (69.37%)	409 (71.13%)	3.27	0.195
	AG	188 (28.79%)	148 (25.74%)		
	GG	12 (1.84%)	18 (3.13%)		
	G	212 (16.23%)	172 (15.11%)	0.57	0.448
rs1367117	GG	496 (75.96%)	434 (75.48%)	0.04	0.981
	AG	147 (22.51%)	132 (22.96%)		
	AA	10 (1.53%)	9 (1.57%)		
	A	167 (12.79%)	150 (13.04%)	0.04	0.850
rs12720838	CC	455 (69.68%)	393 (68.35%)	9.70	0.008
	CT	192 (29.4%)	162 (28.17%)		
	TT	6 (0.92%)	20 (3.48%)		
	T	204 (15.62%)	202 (17.57%)	1.68	0.195
***PCSK9*** **gene**
rs662145	TT	505 (77.34%)	434 (75.48%)	1.34	0.511
	CT	137 (20.98%)	134 (23.30%)		
	CC	11 (1.68%)	7 (1.22%)		
	C	159 (12.17%)	148 (12.87%)	0.27	0.603
rs45448095	CC	544 (83.31%)	454 (78.96%)	3.84	0.147
	CT	103 (15.77%)	115 (20.00%)		
	TT	6 (0.92%)	6 (1.04%)		
	T	115 (8.81%)	127 (11.04%)	3.45	0.063

*SNPs, single nucleotide polymorphisms*.

* *P was calculated using the χ^2^-test*.

### Single-Locus Analysis

The risk prediction of the remaining 6 SNPs for DKD risk under both additive and dominant models is provided in Table 3. After adjusting for confounding factors, including age, gender, smoking, BMI, hypertension, and duration of diabetes, the mutations of rs1042034 and rs12720838 were associated with the significantly increased risk of DKD under both models of inheritance. For instance, carriers of rs1042034 T allele (CT and TT genotypes) were 1.07 times more likely to have DKD than carriers of rs1042034 CC genotype, independent of these confounders (OR = 1.07, 95% CI: 1.03–1.10, *P* < 0.001).

**Table 3:** Risk prediction of 8 studied polymorphisms for diabetic kidney disease under both additive and dominant models.

**SNPs**	**Additive model**		**Dominant model**	
	**OR (95% CI)**	***P***	**OR (95% CI)**	***P***
**Before adjustment**
rs1042034	1.06 (1.03, 1.10)	<0.001	1.06 (1.03, 1.10)	<0.001
rs679899	1.02 (0.99, 1.05)	0.257	1.02 (0.99, 1.05)	0.257
rs1367117	1.00 (0.99, 1.01)	0.845	1.00 (0.99, 1.01)	0.845
rs12720838	1.06 (1.02, 1.10)	0.005	1.06 (1.02, 1.1)	0.005
rs662145	1.00 (0.98, 1.01)	0.459	1.00 (0.98, 1.01)	0.459
rs45448095	1.02 (0.97, 1.08)	0.391	1.02 (0.97, 1.08)	0.391
After adjustment^*^
rs1042034	1.07 (1.03, 1.10)	<0.001	1.07 (1.03, 1.10)	<0.001
rs679899	1.02 (0.99, 1.05)	0.287	1.02 (0.99, 1.05)	0.287
rs1367117	1.00 (0.99, 1.01)	0.883	1.00 (0.99, 1.01)	0.883
rs12720838	1.06 (1.02, 1.11)	0.002	1.06 (1.02, 1.11)	0.002
rs662145	0.99 (0.98, 1.01)	0.334	0.99 (0.98, 1.01)	0.334
rs45448095	1.02 (0.97, 1.07)	0.506	1.02 (0.97, 1.07)	0.506

*SNPs, single nucleotide polymorphisms; OR, odds ratio; 95% CI, 95% confidence interval*.

* *Adjusting for age, gender, smoking, body mass index, hypertension, and duration of diabetes*.

### Haplotype Analysis

Table 4 shows the estimated frequencies of haplotypes separately in *ApoB* and *PCSK9* genes, as well as their prediction for DKD risk. In *ApoB* gene, the most common haplotype was C-A-G-C (alleles in order of rs1042034, rs679899, rs1367117, and rs12720838), and its frequency was 66.54% in controls and 60.90% in cases. In *PCSK9* gene, the most common haplotype was T-C (alleles in order of rs662145 and rs45448095), and its frequency was 80.19% in controls and 82.28% in cases. Comparison of haplotypes between the two groups revealed that haplotype T-A-G-T in *ApoB* gene was overrepresented in cases (18.10%) compared with controls (12.76%) (Simulated *P* = 0.045), and haplotype T-A-G-T was associated with a 33% increased risk of DKD (OR = 1.33, 95% CI: 1.04, 1.70). The power to detect the significant association of haplotype T-A-G-T with DKD was estimated to be 79.9%.

**Table 4:** The frequency of derived haplotypes between controls and cases, and their risk prediction for diabetic kidney disease.

Haplotype^*^	**Controls (%)**	**Cases (%)**	**Hap. score**	***P***	**Simulated *P***	**OR (95% CI)**
***ApoB*** **gene**
C-A-G-C	66.54	60.90	−1.39	0.163	0.175	Reference
T-A-G-T	12.76	18.10	1.81	0.042	0.045	1.33 (1.04, 1.70)
C-G-A-C	6.28	4.64	−0.83	0.408	0.410	0.94 (0.66, 1.35)
T-G-A-C	4.35	4.92	0.68	0.497	0.530	1.22 (0.82, 1.81)
T-G-G-C	2.99	3.23	0.32	0.749	0.700	1.14 (0.70, 1.87)
C-G-G-C	1.84	2.21	−0.10	0.924	0.925	1.22 (0.64, 2.35)
C-A-A-C	1.34	2.49	1.24	0.213	0.210	1.94 (0.97, 3.89)
C-A-G-T	1.64	1.46	−0.62	0.537	0.555	0.94 (0.47, 1.89)
T-A-G-C	0.98	1.05	0.13	0.900	0.920	1.15 (0.49, 2.69)
***PCSK9*** **gene**
T-C	80.19	82.28	−1.34712	0.178	0.185	Reference
C-C	8.77	8.91	−0.13257	0.895	0.885	1.01 (0.75, 1.35)
T-T	6.94	5.54	1.47107	0.141	0.105	1.28 (0.91, 1.8)
C-T	4.10	3.26	1.20819	0.227	0.210	1.3 (0.82, 2.06)

*OR, odds ratio; 95% CI, 95% confidence interval*.

* *In ApoB gene, alleles in a haplotype are assigned in the order of rs1042034, rs679899, rs1367117, and rs12720838; in PCSK9 gene, alleles in a haplotype are assigned in the order of rs662145 and rs45448095*.

No significance was noted for the comparisons of other haplotypes and risk predictions.

### Haplotype-Phenotype Analysis

Further, an analysis on the association of haplotypes as a whole with baseline characteristics was done in both genes (Table 5). Of all characteristics, significant association was only noted for hypertension and omnibus haplotypes in *ApoB* gene (Simulated *P* = 0.001).

**Table 5:** Association of all derived haplotypes as a whole in each gene with baseline characteristics of study subjects.

**Characteristics**	***ApoB*** **gene**			***PCSK9*** **gene**		
	**Global statistics**	***P***	**Simulated *P***	**Global statistics**	***P***	**Simulated *P***
Age	13.08	0.442	0.269	2.57	0.462	0.451
Gender	24.27	0.029	0.069	6.00	0.112	0.104
BMI	8.93	0.778	0.497	1.46	0.692	0.698
Duration of diabetes	11.72	0.468	0.307	1.28	0.734	0.759
Hypertension	104.82	<0.001	0.001	1.12	0.773	0.794
Smoking	13.81	0.387	0.283	0.78	0.854	0.862
TC	12.14	0.516	0.257	1.37	0.712	0.709
HbA1C	17.48	0.178	0.101	0.29	0.963	0.962
HCY	18.18	0.151	0.057	1.61	0.656	0.651
TG	9.51	0.733	0.371	0.44	0.933	0.950
HDLC	6.61	0.921	0.656	2.43	0.488	0.507
LDLC	13.25	0.429	0.210	2.69	0.443	0.407

*BMI, body mass index; TC, total cholesterol; HbA1C, hemoglobin A1c; HCY, homocysteine; TG, triglyceride; HDLC, high-density lipoprotein cholesterol; LDLC, low-density lipoprotein cholesterol*.

## Discussion

To the best of our knowledge, this is the first study that has explored the susceptibility of *ApoB* and *PCSK9* genetic alternations to DKD risk in a large Chinese diabetic population. The key finding of this study is that *ApoB* gene is a candidate gene for DKD. Particularly, two SNPs, rs1042034 and rs12720838, in *ApoB* gene were individually associated with the significant risk of DKD under both additive and dominant models of inheritance, and in the presence of other two SNPs in this gene, the risk was clearly enhanced, indicating the possible synergistic contribution.

DKD is a polygenic disease (18). A long list of genes have been identified to play a contributory role in the pathogenesis of DKD, such as *ADIPOQ* gene (19) and *IL-6* gene (20). However, the results are not often reproducible at a population level. The reasons behind this poor reproducibility are manifold. The most possible reason is the divergence in the genetic underpinnings of different origins of populations. For instance, a polymorphism may be in close linkage with another nearby causal locus in one ethnic group but not in another (21). As such, it is necessary to establish a candidate list of culprit genes and mutations in each ethnic group. Another reason lies in the fact that the net impact of a single gene or single mutation on complex diseases such as DKD may be small or moderate, or its impact may be offset or antagonized by other cellular regulators (22). To shed some light on this issue, besides single-locus analysis, we also interrogated the contribution of unlinked SNPs as a haplotype, and importantly, the risk magnitude in *ApoB* gene was reinforced in haplotype analysis. In particular, the significant haplotype T-A-G-T in *ApoB* gene, harboring the mutant alleles of both rs1042034 and rs12720838, was associated with over 30% increased risk of DKD, in comparison with the increased risk of both SNPs at 6 and 6%, respectively.

The third possible reason is the involvement of environmental or intermediate phenotypes in the development of DKD, such as hypertension, which has been established as a promising risk factor for DKD (23, 24). As expected, we have observed a significant association between omnibus haplotypes in *ApoB* gene and hypertension. Dozens of studies have evaluated the genetic susceptibility of *ApoB* gene to hypertension (25–27). In view of our haplotype-disease and haplotype-phenotype analyses, it is reasonable to speculate that the association of *ApoB* gene with DKD may be mediated by its association with hypertension, which further precipitates the development of DKD. We agree that further studies exploring the concurrent association of *ApoB* genetic defects with hypertension and DKD are needed to confirm or refute this speculation.

Several limitations should be acknowledged for this association study. First, this study is cross-sectional and hospital-based in design, and all study subjects were diagnosed to have type 2 diabetes mellitus. It is of added interest to enroll healthy subjects free of type 2 diabetes mellitus as the controls. Second, only 8 candidate SNPs were selected in *ApoB* and *PCSK9* genes. Third, the sample size is insufficient for further subsidiary investigations, such as upon stratification by gender and hypertension. Fourth, all study subjects are exclusively Chinese, and it leaves an open question for the generalizability of our findings to other ethnic groups.

### Conclusions

Despite these limitations, our findings indicate that *ApoB* gene is a candidate gene for DKD in Chinese patients with type 2 diabetes mellitus. Although no hint of association was detected for *PCSK9* gene, its candidacy in the development of DKD cannot be excluded, and is subject to a matter of debate. For practical reasons, further studies are needed to investigate the underlying mechanisms of ApoB, *in vitro* or *in vivo*, in the pathogenesis of DKD.

## Data Availability Statement

The original contributions generated in the study are included in the article/Supplementary Materials, further inquiries can be directed to the corresponding authors.

## Ethics Statement

The studies involving human participants were reviewed and approved by Institutional Review Boards of the China-Japan Friendship Hospital. The patients/participants provided their written informed consent to participate in this study.

## Author Contributions

LM, YC, PL, and WN: conceptualization. LM, SW, WN, HZ, XD, YJ, and MY: data collection. LM: funding acquisition. LM, SW, and YJ: investigation. LM, HZ, and XD: data detection. WN: statistics. WN, LM, YC, and PL: writing. All authors read and approved the final manuscript prior to submission.

## Conflict of Interest

The authors declare that the research was conducted in the absence of any commercial or financial relationships that could be construed as a potential conflict of interest.
