# Supplementary material for: Susceptibility of ApoB and PCSK9 Genetic Polymorphisms to Diabetic Kidney Disease Among Chinese Diabetic Patients
Source: Front Med (Lausanne). 2021 Apr 6;8:659188. doi: 10.3389/fmed.2021.659188 (PMC8055819; doi:10.3389/fmed.2021.659188)
Supplement: Supplementary file 1 [file Data_Sheet_1.PDF]

Supplementary Table 1. The primer sequences of each single nucleotide polymorphism (SNP) under study.

**SNPs**	**Forward primer**	**Reverse primer**
rs1042034	TTAAGGCATAGGTTTTCTTTC	GCTGAAAGAGATGAAATTTACTT
rs679899	TAGAGTTGATAGTTCCGAGAG	AAAGGATAATAACATGGTGTGT
rs676210	GAATACTGTATAGCTTGCCAAA	CCAACTCTCAACCTTAATGATT
rs1367117	CTCCTCAGAGTTCTTGGTT	GCTCTGCTACCCTGAATT
rs12720838	AGTTTGCCAAATACTTTCTT	CTCCTGACCTCAAGTGAT
rs662145	CTGATTAATGGAGGCTTAGC	CTGGCATAGAGCAGAGTAA
rs45448095	CCCTGCTCCTGAACTTCA	GAGAGGTTGCTGTCCTGG
rs11583680	GACTACGAGGAGCTGGTG	CCTCCCATCCCTACACCC

Supplementary Table 2. The probe sequences of each single nucleotide polymorphism (SNP) under study.

SNPs	Probe sequences
rs1042034-TC	TTAAAAACATATGGGATATAATCAC
rs1042034-TT	ctgTTAAAAACATATGGGATATAATCAT
rs1042034-TR	TGAAGATTGTGTTGATCTCATCTTG
rs679899-TA	ctgaGTTGGAAGTTGAGATTCTTTCAGAA
rs679899-TG	ctgactgGTTGGAAGTTGAGATTCTTTCAGAG
rs679899-TR	CTTCTTTCACTAACTTTTTCAGACTctg
rs676210-TA	ctgactgaGAGATGTGGGGAAGCTGGAATTCTA
rs676210-TG	ctgactgactgGAGATGTGGGGAAGCTGGAATTCTG
rs676210-TR	GTATGTGAAGGTCAGGAACTTGAAActgact
rs1367117-TA	ctgactgactgaTTGAAGCCATACACCTCTTTCAGGA
rs1367117-TG	ctgactgactgactgTTGAAGCCATACACCTCTTTCAGGG
rs1367117-TR	TGCACTGGCTGGTCTTCAGGATGAActgactgac
rs12720838-TC	TCAATCTTAAAAGGACCTTCAGGTC
rs12720838-TT	ctgTCAATCTTAAAAGGACCTTCAGGTT
rs12720838-TR	AGGTGCAGTGGCTCACACCTGTTAT
rs662145-TC	ctgaCTAGCCAGAGGCTGGAGACAGGTGC
rs662145-TT	ctgactgCTAGCCAGAGGCTGGAGACAGGTGT
rs662145-TR	GCCCCTGGTGGTCACAGGCTGTGCCctg
rs45448095-TC	ctgactgaCCACCGCAAGGCTCAAGGCGCCGCC
rs45448095-TT	ctgactgactgCCACCGCAAGGCTCAAGGCGCCGCT
rs45448095-TR	GGCGTGGACCGCGCACGGCCTCTAGctgact
rs11583680-TC	ctgactgactgaTGCGTTCCGAGGAGGACGGCCTGGC
rs11583680-TT	ctgactgactgactgTGCGTTCCGAGGAGGACGGCCTGGT
rs11583680-TR	CGAAGCACCCGAGCACGGAACCACActgactgac
